# Low T3 syndrome predicts more adverse events in patients with hypertrophic cardiomyopathy

**DOI:** 10.1002/clc.24156

**Published:** 2023-09-15

**Authors:** Chao‐Jie He, Chun‐Yan Zhu, Hong‐Yan Fan, Ye‐Zhou Qian, Chang‐Lin Zhai, Hui‐Lin Hu

**Affiliations:** ^1^ Department of Cardiology The Affiliated Hospital of Jiaxing University Jiaxing Zhejiang China; ^2^ Department of Anesthesiology The Affiliated Hospital of Jiaxing University Jiaxing Zhejiang China

**Keywords:** heart failure, hypertrophic cardiomyopathy, low T3 syndrome, sudden cardiac death

## Abstract

**Background:**

Hypertrophic cardiomyopathy (HCM) is a common cardiac genetic disorder that clinically manifests with sudden death and progressive heart failure. Moreover, thyroid dysfunction is associated with increased cardiovascular morbidity and mortality risks. Therefore, this study aimed to clarify whether thyroid hormones could serve as an independent predictor of adverse events in patients with HCM.

**Methods:**

The cohort consisted of 782 patients with HCM who had thyroid hormones baseline data and were admitted to the Affiliated Hospital of Jiaxing University. Patients were divided into two groups according to serum levels of free triiodothyronine (fT3): the normal fT3 and low triiodothyronine (T3) syndrome groups. Low T3 syndrome was defined as fT3 < 2.43 pmol/L with a normal thyroid‐stimulating hormone (TSH) level. Patients whose TSH levels were abnormally high or abnormally low were excluded from this study. The primary endpoint was the occurrence of sudden cardiac death (SCD) events, and the secondary endpoint was a composite of worsening heart failure (WHF) events, including heart failure death, cardiac decompensation, hospitalization for heart failure, and HCM‐related stroke. The Kaplan–Meier and Cox regression were performed for the survival analysis.

**Results:**

After a median follow‐up of 52 months, 75 SCD events and 134 WHF events were recorded. The Kaplan–Meier survival curves showed that the cumulative incidence of SCD events and WHF events were significantly higher in patients with low T3 syndrome (log‐rank *p* = .02 and log‐rank *p* = .001, respectively). Furthermore, multivariate Cox regression analysis demonstrated that low T3 syndrome is a strong predictor of SCD events and WHF events (adjusted hazard ratio [HR: 1.53, 95% confidence interval [CI]: 1.13–2.24, *p* < .01; HR: 3.87, 95% CI: 2.91–4.98, *p* < .001, respectively).

**Conclusions:**

Low T3 syndrome is highly prevalent among patients with HCM and was independently associated with an increased risk of SCD events and WHF events. The routine assessment of serum fT3 levels may provide risk stratification in this population.

## Graphical abstract

Low triiodothyronine (T3) syndrome is highly prevalent among patients with hypertrophic cardiomyopathy and was independently associated with an increased risk of sudden cardiac death events and worsening heart failure events.

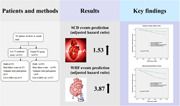

## 1 INTRODUCTION

Hypertrophic cardiomyopathy (HCM) is the most common inherited primary cardiomyopathy that affects approximately 1 in 500 individuals.^1^, ^2^ Pathologically, it manifests as left ventricular hypertrophy, myofibrillar disarray, fibrosis, and arterial remodeling.^3^, ^4^ Sudden cardiac death (SCD) and progression toward end‐stage heart failure are two major causes of death in this population.^5^ Several clinical characteristics have been identified as prognostic factors for adverse outcomes, including maximum left ventricular wall thickness (MLVWT) of >30 mm, left atrial enlargement, family history of SCD, unexplained syncope, nonsustained ventricular tachycardia (VT), and left ventricular outflow tract obstruction (LVOTO).^6^, ^7^, ^8^

Thyroid hormones act as a fundamental regulator of cardiac function.^9^ Over the past two decades, evidence has demonstrated an association between abnormal thyroid status in patients with cardiovascular disease and subsequent poor outcomes, particularly the level of free triiodothyronine (fT3).^10^, ^11^ Meanwhile, thyroid hormones are affected by severe cardiac conditions, including myocardial infarction, heart failure, acute myocarditis, and acute ischemic stroke, and abnormal levels of thyroid hormones may serve as a potential biomarker of poor prognosis.^12^, ^13^

Low triiodothyronine (T3) syndrome, also known as nonthyroidal illness syndrome or euthyroid sick syndrome, is characterized by decreased plasma fT3 concentrations without elevated thyroid‐stimulating hormone (TSH) levels.^14^ Lower serum fT3 levels may be detrimental to cardiovascular homeostasis because of its crucial role in regulating cardiac contractility, maintaining mitochondrial integrity and cardiac function, modulating systemic vascular resistance, and postischemic left ventricular remodeling.^15^, ^16^

The relationship between thyroid dysfunction and psychiatric disorders, particularly depression and anxiety, has been well acknowledged.^17^ Patients with thyroid disease are more predisposed to depressed mood; in contrast, individuals with depressive symptoms may also have subtle thyroid dysfunction.^18^ Our prior work, combined with other clinical findings, demonstrated that depression was a significant predictor of cardiovascular adverse events in patients suffering from HCM.^19^ However, the impact of fT3 levels on the long‐term prognosis in patients with HCM and whether low T3 syndrome is a predictor of SCD events and heart failure events in this population have not been examined. Hence, this study aimed to investigate the prevalence of low T3 syndrome and the association between low T3 syndrome and clinical outcomes in patients with HCM.

## 2 METHODS

### 2.1 Study population and design

Between February 2014 and March 2018, we performed a prospective observational study of 782 patients diagnosed with HCM from either the outpatient department or inpatient wards of the Affiliated Hospital of Jiaxing University.

The main inclusion criteria were that the participants met the diagnosis of HCM according to the guidelines of the European Society of Cardiology (ESC): (1) maximum wall thickness ≥15 mm in at least one left ventricular myocardial segment, as measured by echocardiography, computed tomography, or cardiac magnetic resonance; (2) wall thickness of 13–14 mm with a definite family history of HCM; and (3) left ventricular wall hypertrophy cannot be explained simply through abnormal loading conditions or flow‐limiting coronary heart disease.^20^ The exclusion criteria were as follows: (1) past or present diagnosis of thyroid disease, including thyroiditis, hypothyroidism, or hyperthyroidism; (2) participants receiving medications that may influence thyroid function (levothyroxine, antithyroid agents, iodine, or corticosteroids); (3) age <18 years; (4) patients who had severe liver or kidney dysfunction or a malignant tumor; (5) individuals who were unable or unwilling to participate; and (6) hypothyroidism or hyperthyroidism.

This study was conducted following the Declaration of Helsinki, which gained approval from the Ethical Review Committee of the Affiliated Hospital of Jiaxing University, Jiaxing University, Zhejiang, China. Each participant provided informed consent before enrollment.

### 2.2 Clinical characteristics

Patients' demographics, medical history, laboratory findings, echocardiographic data, prescribed medications at discharge, and New York Heart Association (NYHA) classification were obtained and verified from the Nanjing Haitai Medical Information System (version 3.0). Additionally, clinical and ultrasound parameters that show a strong association with the risk of SCD in the HCM population, including a family history of sudden death, unexplained syncope, nonsustained VT, MLVWT, and LVOTO, were obtained.^21^ Patients who had an estimated 5‐year risk of sudden deaths of at least 6% according to the ESC guidelines or a history of cardiac arrest were recommended for the implantation of an implantable cardioverter defibrillator (ICD).^20^

### 2.3 Diagnosis of low T3 syndrome

**Figure 1:**
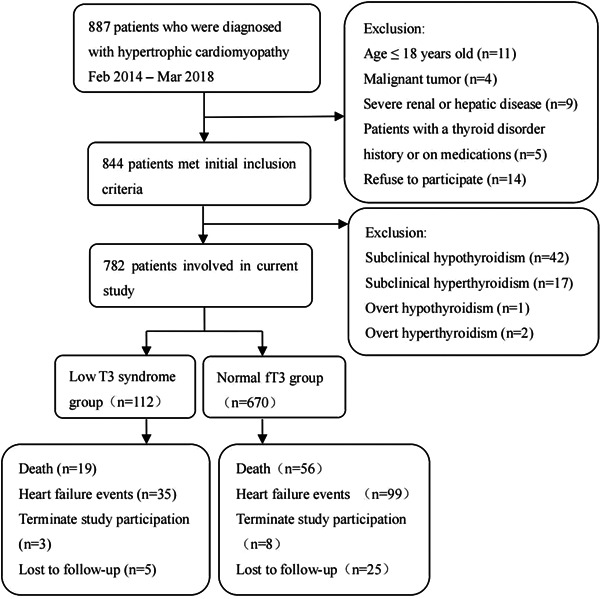
The flowchart of the participant enrollment and screening procedures of study cohort.

The thyroid status was detected by measuring serum levels of fT3, free thyroxine (fT4), and TSH using an enhanced chemiluminescence method (I4000; Abbott Laboratories). In our laboratory, the reference intervals of thyroid hormones and TSH were as follows: fT3: 2.43–6.02 pmol/L, fT4: 9.01–19.03 pmol/L, and TSH: 0.36–4.95 μIU/mL. We first excluded patients with hypothyroidism or hyperthyroidism based on the combination of TSH and fT4 serum levels: subclinical hypothyroidism (TSH > 4.95 μIU/mL, normal fT3 and fT4, *n* = 42), subclinical hyperthyroidism (TSH < 0.36 μIU/mL, normal fT3 and fT4, *n* = 17), overt hypothyroidism (TSH > 4.95 μIU/mL and fT4 < 9.01 pmol/L, *n* = 1), overt hyperthyroidism (TSH < 0.36 μIU/mL and fT4 > 19.03 pmol/L, *n* = 2; Figure 1).

Low T3 syndrome was described as serum fT3 levels <2.43 pmol/L, with fT4 and TSH within the normal range but usually borderline in patients with euthyroid status. No further treatment for low T3 syndrome was recommended owing to the lack of evidence supporting the effectiveness and safety of levothyroxine supplements in this population. According to the fT3 level on admission, the participants were divided into the normal fT3 group (fT3: 2.43–6.02 pmol/L) and the low T3 syndrome group (fT3 < 2.43 pmol/L). There was no patient whose TSH values were normal with fT3 values elevated (>6.02 pmol/L) in the present study.

### 2.4 Primary and secondary endpoints

The participants were followed up through subsequent outpatient visits, telephone contact, and WeChat every 3 months for the first year and then every 6 months for the remaining period after discharge. Survival data and heart failure events were recorded by trained cardiologists at the Affiliated Hospital of Jiaxing University. Those who were lost to follow‐up were censored at their last interview.

The primary endpoint of this study was SCD events, defined as sudden death due to cardiac causes, ICD shocks, or recorded cardiac arrest.^22^ SCD was described as an unexpected death within 60 min after the onset of cardiac symptoms and nocturnal deaths without any antecedent history of worsening symptoms or confirmed by autopsy. Recorded cardiac arrest was characterized as ventricular fibrillation or VT during Holter or ICD monitoring. Furthermore, ICD shocks in which patients were either successfully resuscitated or received appropriate defibrillation were considered to be equivalent to SCD events in the present data analysis. Worsening heart failure (WHF) events were prespecified secondary endpoints, described as a composite of heart failure death, cardiac decompensation, hospitalization for heart failure, and HCM‐related stroke. Cardiac decompensation was defined as participants with cardiac function in class I or II that progressed to class III or worse, in accordance with the NYHA functional class.^23^ The definition of each adverse event is comprehensively described in our former article.^19^

### 2.5 Statistical analysis

Continuous data were presented as mean ± standard deviation and analyzed by the Student *t*‐test. Categorical data were summarized as percentages, and differences were compared using the *χ* ^2^ test. Moreover, Fisher's exact test was applied when assumptions for the *χ* ^2^ test were invalid. Survival analysis was performed using Kaplan–Meier survival curves, and log‐rank tests were employed to compare differences between patients with normal fT3 and low T3. Univariate and multivariate proportional analyses were used using the Cox proportional hazard regression model to examine the relationship between low T3 syndrome and adverse events during a median follow‐up of 52 months.

The stepwise model selection method was used for prognostic variable selection. The significance level required for an entry criterion was <0.20, and the exit probability was >0.05. Moreover, a forced entry strategy was employed, and a priori established SCD risk factors were entered into the multivariate model. As a result, variables that entered the final model for SCD events include unexplained syncope, family history of sudden death, MLVWT > 30 mm, nonsustained VT on Holter monitoring, LVOTO, and depression. After the prespecified model was developed, other confounding variables (i.e., β‐blockers and fT4) were retested based on a sensitivity analysis to verify their influence on effect estimates. Multivariate models were established individually for SCD events and WHF events in a similar approach. Crude and adjusted hazard ratios (HRs) were calculated with 95% confidence intervals (CIs). *p* < .05 was considered statistically significant. All statistical analyses were conducted using IBM SPSS Statistics for Windows version 24.0 (IBM Corp.).

## 3 RESULTS

### 3.1 Baseline characteristics

The study cohort included 782 patients with HCM aged 49.9 ± 13.1 years, of whom 58.4% were men. Among these, 112 (14.3%) were in the low T3 syndrome group, and 670 (85.7%) were in the normal fT3 group. Moreover, 741 participants (94.8%) completed a median follow‐up of 52 months, and both groups exhibited comparable dropout rates (Figure 1). Table 1 shows the baseline characteristics, laboratory findings, echocardiographic parameters, and serum thyroid hormone and TSH levels of the study population. Patients who had lower fT3 were more likely to be men and have a history of stroke, higher probrain natriuretic peptide levels, and worse NYHA classification at baseline. As expected, the low T3 syndrome group had lower fT4 and higher TSH levels. No other statistically significant differences were observed between the two groups. Additionally, 346 participants with HCM had at least one or more traditional risk factors for SCD, whereas merely 10% of the participants had undergone ICD implantation.

**Table 1:** Baseline characteristics of the study population with or without LT3S.

Characteristics	LT3S (*n* = 112)	Normal fT3 (*n* = 670)	*p*‐Value
Demographics			
Age (mean ± SD) year	50.3 ± 13.7	49.7 ± 12.8	.083
Male, *n* (%)	57 (50.9)	400 (59.7)	.002
BMI (mean ± SD) kg/m^2^	24.3 ± 3.6	24.7 ± 3.9	.575
Maximum LV thickness (mean ± SD) mm	20.2 ± 4.8	20.1 ± 4.6	.699
NYHA class, *n* (%)			
I/II	97 (86.6)	589 (87.9)	.07
III/IV	15 (13.4)	81 (12.1)	.07
Comorbid condition, *n* (%)			
Atrial fibrillation	11 (9.8)	65 (9.7)	.636
Stroke history	3 (2.7)	6 (0.9)	.032
Diabetes	14 (12.5)	84 (12.5)	.697
Hypertension	20 (17.9)	106 (15.8)	.721
Sudden death risk factors, *n* (%)			
Nonsustained VT on Holter	20 (17.9)	116 (17.3)	.452
Unexplained syncope	11 (9.8)	54 (8.1)	.135
Family history of SCD	14 (12.5)	82 (12.2)	.514
LVOTO	23 (20.5)	147 (21.9)	.254
MLVWT ≥ 30 mm	6 (5.4)	32 (4.8)	.633
Echocardiography (mean ± SD)			
LVEF (%)	61.7 ± 6.1	63.1 ± 6.2	.124
Left atrial diameter	40.2 ± 9.2	39.9 ± 9.1	.323
ICD implantation			
ICD, *n* (%)	12 (10.7)	62 (9.3)	.123
Medications at discharge, *n* (%)			
β‐Blockers	69 (61.6)	428 (63.9)	.453
Calcium channel blockers	21 (18.8)	118 (17.6)	.091
ACEI or ARB	20 (17.9)	107 (16.0)	.103
Diuretic	10 (8.9)	48 (7.2)	.113
Amiodarone	3 (2.7)	19 (2.8)	.636
Laboratory findings (mean ± SD)			
fT3 (pmol/L)	2.2 ± 0.2	4.3 ± 0.7	<.001
fT4 (pmol/L)	12.0 ± 3.6	13.9 ± 3.9	.008
TSH (μIU/ml)	4.1 ± 0.7	2.2 ± 0.5	<.001
Pro‐BNP (pg/mL)	729.8 ± 493.1	579.1 ± 466.3	.003
Creatinine (µmol/L)	76.2 ± 13.4	75.3 ± 13.3	.363

Abbreviations: ACEI, angiotensin‐converting enzyme inhibitors; ARB, angiotensin receptor blockers; BMI, body mass index; fT3, free triiodothyronine; fT4, free thyroxine; ICD, implantable cardioverter defibrillator; LT3S, low triiodothyronine syndrome; LV, left ventricle; LVEF; left ventricular ejection fraction; LVOTO, left ventricular outflow tract obstruction; MLVWT, maximum left ventricle wall thickness; NYHA, New York Heart Association; Pro‐BNP, probrain natriuretic peptide; SCD, sudden cardiac death; SD, standard deviation; THS, thyroid‐stimulating hormone; VT, ventricular tachycardia.

### 3.2 Clinical outcomes

During the follow‐up, 75 patients with euthyroid with HCM suffered from an SCD event (sudden death: 39; cardiac arrest: eight, and ICD shock: 28), and 134 developed WHF events (deaths caused by heart failure: 15, hospitalizations for heart failure: 23, cardiac transplantation: 1, HCM‐related stroke: 17, and progressive heart failure symptoms to NYHA classes III and IV: 78) (Supporting Information: Table S1). Of 19 SCD patients with low T3 syndrome, 7 (36.8%) had no conventional risk factors for SCD, and 12 (63.2%) had at least one risk factor. Within the follow‐up period, 10 died suddenly, two were revived from an aborted cardiac arrest, and seven received appropriate ICD discharge.

### 3.3 Association between low T3 syndrome and clinical outcomes

**Figure 2:**
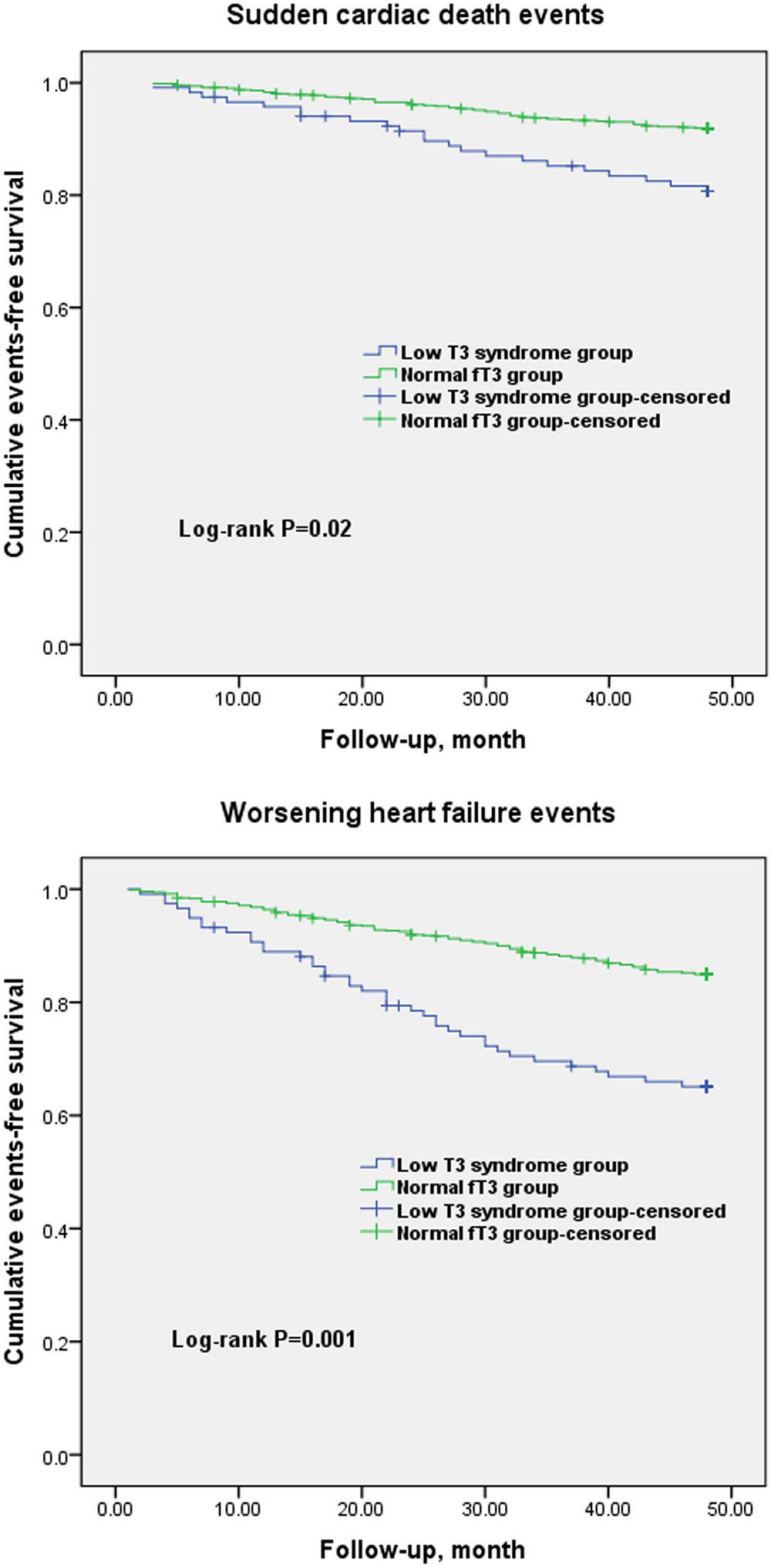
Kaplan–Meier survival curves for sudden cardiac death events and worsening heart failure events in patients with or without low T3 syndrome.

The Kaplan–Meier survival curves indicated that the cumulative incidence of SCD events (log‐rank *p* = .02; Figure 2) and WHF events (log‐rank *p* = .001) was significantly increased in patients with low T3 syndrome than in the normal fT3 group. Table 2 shows the results of the univariate Cox survival analysis for adverse outcomes. In thyroid function profiles, a low fT3 level was found to be a significant predictor of SCD events and WHF events (HR = 1.63, 95% CI: 1.20–2.49, *p* < .01; HR = 2.62, 95% CI: 2.03–3.49, *p* < .001, respectively). Two multivariate models were constructed individually to investigate the effects of low fT3 levels on SCD events and WHF events. After adjusting for traditional risk factors and depression, multivariate Cox regression demonstrated that low T3 syndrome remained an independent predictor of SCD events and WHF events (adjusted HR = 1.53, 95% CI: 1.13–2.24, *p* < .01; HR = 3.87, 95% CI: 2.91–4.98, *p* < .001, respectively; Table 3). In accordance with previously published findings, family history of sudden death, unexplained syncope, LVOTO, nonsustained VT on 24‐h Holter monitoring, and MLVWT > 30 mm were further associated with a poor prognosis in this analysis. Notably, patients with low T3 syndrome showed an approximately fourfold higher risk of WHF events after adjustment. To be noted, our findings indicated that depression predicted SCD events and WHF events in patients with HCM, which is generally consistent with our previous work.^19^

**Table 2:** Univariate Cox regression of variables influencing SCD events and WHF events.

Variables	SCD events		Worsening heart failure events	
	HR (95% CI)	*p*‐Value	HR (95% CI)	*p*‐Value
Age (per decade increase)	0.97 (0.86–1.08)	.462	1.07 (0.97–1.17)	.123
Left atrial diameter	1.05 (0.83–1.11)	.269	1.11 (0.82–1.21)	.096
Atrial fibrillation	1.01 (0.83–1.11)	.869	1.28 (1.08–1.68)	.147
β‐Blockers	0.74 (0.63–0.93)	.019	0.83 (0.69–0.94)	.023
Calcium channel blockers	0.93 (0.80–1.02)	.365	0.96 (0.85–1.12)	.442
RAAS inhibitors	0.97 (0.99–1.05)	.636	0.85 (0.75–1.08)	.083
Diuretic	1.03 (0.97–1.08)	.712	0.91 (0.82–1.13)	.451
NSVT on ambulatory Holter	2.86 (2.36–3.61)	<.001	1.49 (1.06–1.96)	<.01
Unexplained syncope	2.01 (1.44–2.80)	.007	1.11 (0.93–1.23)	.596
Family history of SCD	1.70 (1.39–2.16)	.029	1.08 (0.92–1.12)	.459
LVOTO	2.22 (1.57–2.89)	<.001	2.35 (1.93–3.53)	<.001
MLVWT ≥ 30 mm	1.31 (1.09–1.75)	.041	1.49 (1.06–2.06)	.029
Depression	2.03 (1.63–2.49)	<.001	1.86 (1.29–2.81)	<.001
fT4	1.09 (0.92–1.33)	.162	1.13 (0.97–1.45)	.084
LT3S	1.63 (1.20–2.49)	<.01	2.62 (2.03–3.49)	<.001

Abbreviations: CI, confidence interval; fT4, free thyroxine; HCM, hypertrophic cardiomyopathy; HR, hazard ratio; LT3S, low triiodothyronine syndrome; LVOTO, left ventricular outflow tract obstruction; MLVWT, maximum left ventricle wall thickness; NSVT, nonsustained ventricular tachycardia; RAAS, rennin–angiotensin–aldosterone system; SCD, sudden cardiac death; WHF, worsening heart failure.

**Table 3:** Multivariate Cox regression of variables influencing SCD events and WHF events.

Variables	SCD events		Worsening heart failure events	
	HR (95% CI)	*p*‐Value	HR (95% CI)	*p*‐Value
β‐Blockers	0.76 (0.65–0.98)	.032	0.82 (0.71–0.99)	.026
Atrial fibrillation	NA	NA	1.16 (0.91–1.28)	.091
RAAS inhibitors	NA	NA	0.92 (0.82–1.21)	.097
fT4	1.07 (0.90–1.32)	.124	1.15 (0.98–1.46)	.072
NVT on ambulatory Holter	2.68 (2.22–3.27)	<.001	1.47 (1.04–1.88)	.027
Unexplained syncope	1.89 (1.42–2.40)	.012	1.11 (0.91–1.22)	.723
Family history of SCD	1.76 (1.37–2.21)	<.01	1.06 (0.90–1.16)	.842
LVOTO	2.16 (1.64–2.62)	<.001	2.22 (1.89–2.79)	<.001
MLVWT ≥ 30 mm	1.21 (1.02–1.48)	.042	1.39 (1.09–1.97)	.002
Depression	1.89 (1.49–2.63)	<.001	1.76 (1.43–2.33)	<.001
LT3S	1.53 (1.13–2.24)	<.01	3.87 (2.91–4.98)	<.001

Abbreviations: CI, confidence interval; fT4, free thyroxine; HCM, hypertrophic cardiomyopathy; HR, hazard ratio; LT3S, low triiodothyronine syndrome; LVOTO, left ventricular outflow tract obstruction; MLVWT, maximum left ventricle wall thickness; NA, not applicable; NVT, nonsustained ventricular tachycardia; RAAS, rennin–angiotensin–aldosterone system; SCD, sudden cardiac death; WHF, worsening heart failure.

## 4 DISCUSSION

To our knowledge, this study is the first to clarify the potential association between low T3 syndrome and adverse events in patients with HCM. Our study finds that low T3 syndrome comprises 14.3% of euthyroid patients with HCM. It shows that low fT3 levels are associated with an increased risk of SCD events and WHF events. In our earlier studies, patients with depression and HCM had more than double the risk of SCD events.^19^ In the current study, we use multivariate Cox regression analysis to adjust for traditional risk factors and depression. In HCM patients, low T3 syndrome correlates with a 1.53 HR for SCD events and a 3.87 HR for WHF events. Depression, by contrast, correlates with a 1.89 HR for SCD events, and a 1.76 HR for WHF events.

Thyroid dysfunction is commonly seen in cardiovascular diseases and serves as a strong predictor of subsequent adverse outcomes. Two decades ago, Iervasi et al.^24^ reported that low serum fT3 is an independent predictor of death in patients with cardiovascular diseases after 1‐year follow‐up. Recently, Su et al.^25^ analyzed 2459 patients with acute myocardial infarction and indicated that low T3 syndrome was associated with more severe myocardial damage and elevated in‐hospital cardiovascular mortality. Additionally, numerous studies have reported that low serum levels of fT3 could predict adverse clinical outcomes in acute and chronic heart failure.^26^, ^27^ According to a recent systematic review and meta‐analysis of 41 studies, the prevalence of low T3 syndrome was 24.5%, 18.9%, and 17.1% in patients with heart failure, acute myocardial infarction, and acute coronary syndrome, respectively.^28^ Herein, low T3 syndrome is commonly seen in the HCM population and has a high prevalence of 14.3%, which is generally consistent with prior findings in the population with cardiovascular disorders. Furthermore, Wang et al.^28^ have concluded that low T3 syndrome was associated with elevated risks of all‐cause mortality, cardiac mortality, and major adverse cardiovascular events.

Currently, clinical studies evaluating the effect of thyroid hormones on patients with HCM are limited. To date, only the study by Zhang et al.^29^ has investigated the relationship between fT3 and hypertrophic obstructive cardiomyopathy (HOCM). Their findings indicated that low fT3 positively correlated with the left ventricular ejection fraction and could be an independent predictor of all‐cause mortality and heart transplantation in this population. However, patients were classified into three groups based on the fT3 levels in their study, and only death and cardiac transplant events were documented during the follow‐up period. Accordingly, another retrospective study recruited 806 patients with HOCM and revealed that decreased serum fT3 levels were associated with the occurrence of atrial fibrillation.^30^ Thyroid abnormalities were observed in 96 patients (11.9%), and subclinical hypothyroidism or subclinical hyperthyroidism accounted for the main proportion (9.6%) of the entire HOCM cohort in a study by Liu et al.^30^ Herein, 14% of the HCM study population had low T3 syndrome and this was significantly associated with increased SCD events and increased WHF events. Our findings revealed that low fT3 levels have prognostic value in patients with HCM apart from conventional risk factors. That is, serum fT3 can provide valuable predictive information for ICD implantation evaluation and risk assessment of heart failure progression.

The potential mechanisms linking low fT3 levels and adverse outcomes have not been completely elucidated; however, several interpretations have been proposed. Generally, both T3 and T4 are crucial for cardioprotection and constitute the major active forms of thyroid hormones; however, T3 is the only bioactive hormone for cardiomyocytes, whereas T4 is believed to be the precursor of T3.^31^ T3 modulates myocardial cell growth, cardiac contractility, and other cardiac functions through genomic and nongenomic pathways.^32^ Other mechanisms, such as endothelial function, vascular resistance, and heart rate, have also been explained.^14^, ^33^ Generally, all these mechanisms collectively contribute to increased risks of cardiovascular events. Additionally, the complex effects of thyroid hormones on the cardiomyocytes, matrix, and ventricular function were confirmed by several animal experiments.^34^, ^35^ However, the pathophysiological processes of low fT3 in euthyroid patients with HCM have not been fully elucidated. Further studies must validate our findings and explore the molecular mechanisms underlying this prognostic association.

Despite the encouraging findings, this study has several limitations. First, this was a single‐center observational study, which limits the generalizability to other populations and could be further confirmed by multicenter studies. Second, thyroid status was measured only at baseline and may have changed in a longer follow‐up period. Third, patients who were prescribed medications that may influence thyroid function were excluded; consequently, the effect of thyroid treatment on clinical outcomes was not explored in this study. Finally, since no intervention was adopted to increase the serum fT3 levels in their normal range, studies should investigate whether fT3 supplements would benefit patients with low T3 syndrome and improve their prognosis in the future. Concurrently, the strengths of our study include a large sample size, a well‐characterized HCM population, comprehensive data on thyroid function, detailed cardiac assessment, an observational study with few patients lost to follow‐up, and adjustment for the traditional risk factors in the multivariate model.

## 5 CONCLUSIONS

Our study demonstrated that low T3 syndrome is highly prevalent among patients with HCM and was associated with SCD events and WHF events. Routine assessment of fT3 levels may provide risk stratification in this population.

## CONFLICT OF INTEREST STATEMENT

The authors declare no conflict of interest.

## Supporting information

Supporting information.

## Data Availability

The raw data supporting the conclusions of this article will be made available by the authors, without undue reservation.
