# Supplementary material for: Low T3 syndrome predicts more adverse events in patients with hypertrophic cardiomyopathy
Source: Clin Cardiol. 2023 Sep 15;46(12):1569–77. doi: 10.1002/clc.24156 (PMC10716344; doi:10.1002/clc.24156)
Supplement: Supplementary file 1 — Supporting information. [file CLC-46-1569-s001.doc]

**Table S1.** Major adverse clinical events of the study population during follow-up

| Major clinical events, n (%) | LT3S (n=112) | Normal fT3 (n=670) |
| --- | --- | --- |
| HCM-related sudden death | 10 (8.9) | 29 (4.3) |
| Documented cardiac arrest | 2 (1.8) | 6 (0.9) |
| ICD shock (VT/VF) | 7 (6.3) | 21 (3.1) |
| Heart failure death | 4 (3.6) | 11 (1.6) |
| Cardiac transplantation | 0 (0) | 1 (0.1) |
| HCM-related stroke | 4 (3.6) | 13 (1.9) |
| Progression to NYHA class Ⅲ/Ⅳ | 22 (17.9) | 56 (7.3) |
| Heart failure hospitalization | 5 (4.5) | 18 (2.7) |

LT3S indicates low triiodothyronine syndrome; fT3, free triiodothyronine; HCM, hypertrophic cardiomyopathy; ICD, implantable cardioverter defibrillator; VT, ventricular tachycardia; VF, ventricular tachycardia; NYHA, New York Heart Association.
